# Characterization of arbuscular mycorrhizal fungal species associating with *Zea mays*

**DOI:** 10.3389/fpls.2024.1345229

**Published:** 2024-05-07

**Authors:** Sílvia Maússe-Sitoe, Joanna Dames

**Affiliations:** Mycorrhizal Research Laboratory, Department of Biochemistry and Microbiology, Rhodes University, Makhanda, South Africa

**Keywords:** AM fungi, maize crop, morphological characterization, single spore, trap culture

## Abstract

Taxonomic identification of arbuscular mycorrhizal (AM) fungal spores extracted directly from the field is sometimes difficult because spores are often degraded or parasitized by other organisms. Single-spore inoculation of a suitable host plant allows for establishing monosporic cultures of AM fungi. This study aimed to propagate AM fungal spores isolated from maize soil using single spores for morphological characterization. First, trap cultures were established to trigger the sporulation of AM fungal species. Second, trap cultures were established with individual morphotypes by picking up only one spore under a dissecting microscope and transferring it to a small triangle of sterilized filter paper, which was then carefully inoculated below a root from germinated sorghum seeds in each pot and covered with a sterile substrate. All pots were placed in sunbags and maintained in a plant growth room for 120 days. Spores obtained from single spore trap cultures from each treatment, maize after oats (MO), maize after maize (MM), maize after peas (MP), and maize after soybean (MS), were extracted using the sieving method. Healthy spores were selected for morphological analysis. Direct PCR was conducted by crushing spores in RNAlater and applying three sets of primer pairs: ITS1 × ITS4, NS31 × AML2, and SSUmcf and LSUmBr. Nucleotide sequences obtained from Sanger sequencing were aligned on MEGA X. The phylogenetic tree showed that the closest neighbors of the propagated AM fungal species belonged to the genera *Claroideoglomus*, *Funneliformis*, *Gigaspora*, *Paraglomus*, and *Rhizophagus*. The morphological characteristics were compared to the descriptive features of described species posted on the INVAM website, and they included *Acaulospora cavernata*, *Diversispora spurca*, *Funneliformis geosporus*, *Funneliformis mosseae*, *Gigaspora clarus*, *Gigaspora margarita*, *Glomus macrosporum*, *Paraglomus occultum*, and *Rhizophagus intraradices*. These findings can provide a great contribution to crop productivity and sustainable management of the agricultural ecosystem. Also, the isolate analyzed could be grouped into efficient promoters of growth and mycorrhization of maize independent of their geographical location.

## 1 Introduction

Mycorrhizal fungi are soil microorganisms distributed worldwide. They form symbiotic and mutualistic associations with more than 80% of vascular land plants (Smith and Read, 2008; Brundrett, 2009). Different types of mycorrhizal associations involving other groups of fungi and host plants have been identified. Among them, arbuscular mycorrhizal (AM) fungi form symbiotic associations with plants (Hata et al., 2010) in almost all habitats and climates (Azcón-Aguilar and Barea, 1997; Chen et al., 2018), including disturbed agricultural soils (Enkhtuya et al., 2000) and those derived from mining activities (Bi et al., 2003). These are considered Earth’s most essential and ubiquitous symbionts (Dodd et al., 1996; Bever et al., 2001). AM fungi are obligate biotrophs that require the roots of a living host to grow and complete their life stages (Kehri et al., 2018). They colonize plant roots in exchange for carbohydrates, up to 20% of photosynthetically fixed, organic carbon (C)-based compounds (C) (Jakobsen and Rosendahl, 1990; Smith and Read, 2008), and provide their plant hosts with mineral nutrients required for plant growth (Jakobsen et al., 1992; Luginbuehl et al., 2017).

The importance of AM fungi is being increasingly considered in agriculture, horticulture, forestry, and environmental reclamation (IJdo et al., 2011; Sheteiwy et al., 2023). The global use of agriculturally beneficial microorganisms tends to contribute directly or indirectly to crop improvement and increases nutrient uptake efficiency (Bargaz et al., 2018; Plett, 2018; El-Sawah et al., 2023). Potentially, AM fungi could replace inorganic fertilizers or at least reduce the use of inorganic fertilizers (Begum et al., 2019; Sheteiwy et al., 2022).

AM fungi form symbiotic relationships with most vascular plants, and identifying AM fungal species is essential. Maize (*Zea mays* L.) is one of the most important crops grown globally for livestock feed, food, and industrial materials (Lv et al., 2016). Maize plants produce high dry matter yields and therefore have a high requirement for nutrients, especially three macro-nutrients: nitrogen (N), phosphorous (P), and potassium (K) (Pettigrew, 2008; Zörb et al., 2014). The use of artificial chemical fertilizers has been an effective way to improve maize production worldwide (Gao et al., 2017; Dai et al., 2018). Although many nutrients required to grow maize are abundant in soil, some may occur at low levels (Mtambanengwe and Mapfumo, 2009; Gao et al., 2020). The majority of plant species form symbiotic relationships with AM fungi to augment N and P uptake from soil (Begum et al., 2019; Yang et al., 2022). AM fungi transfer N (~20%) and P (~90%) to plants in exchange for C from photosynthates (Smith and Read, 2002; Whiteside et al., 2009). It can also boost plant growth by an average of 80% under unfertilized conditions (Hoeksema et al., 2010). In many parts of the world, maize production occurs in semi-arid environments and thus often faces high temperatures and water scarcity (Cairns et al., 2012; Zhao et al., 2016; He et al., 2017). These climate change-induced stresses have significantly threatened maize yields and decreased world maize production by 15%–20% annually (Lobell et al., 2011; Chen et al., 2012; Abdoulaye et al., 2019).

Traditionally, microscopic examination of extracted spores was used to identify AM fungal species before molecular techniques were established (Sanders, 2004; Young, 2012). Conventional morphological observation is still essential and should be addressed for identification, although there is a trend that the sequence data of AM fungi are over-emphasized for identification. The recommendation is that molecular and morphological characterization should be combined where practical because of the production of unique and crucial information, such as the impact of land management on AM fungal spore abundance and richness from each of the methods (Overby et al., 2015; Säle et al., 2015).

Taxonomic identification of AM fungal spores extracted directly from the field is sometimes difficult because spores are often degraded or parasitized by other organisms (Clapp et al., 1995), and it is recommended that morphological spore characteristics are best observed in trap cultures. The Glomeromycota classification based on morphological and molecular data was revised in 2013 by systematists with expertise in the biology and taxonomy of AM fungi (Redecker et al., 2013). The taxonomic classification of AM fungi was constructed by grouping the fungal strains based on similarities and differences in their morphological characteristics (spore morphology, spore formation, and spore wall structure) (Gerdemann and Trappe, 1974; Walker and Sanders, 1986; Morton and Benny, 1990; Schenck and Perez, 1990). Most of the 214 currently described species (https://www.amf-phylogeny.com) are characterized only by spore morphology, and the majority of older species have not been cultured *in vitro* (trap cultures) (Tisserant et al., 1998; Clapp et al., 2001). There are approximately 160 species of AM fungi described by spore morphology according to the International Collection of Vesicular Arbuscular Mycorrhizal Fungi (INVAM; http://invam.caf.wvu.edu/). The variation in morphological characteristics in the spores of AM fungi is limited, thus creating difficulties in identification and morphotyping (Tisserant et al., 1998; Clapp et al., 2001). Walker and Vestberg (1998) reported that in nature there can be significant variation in spore morphology even within an AM fungal species. Still, many AM fungi may reproduce only vegetatively without producing spores (Helgason et al., 2002). Also, fungal spore diversity differs seasonally, with some fungi sporulating in late spring and others at the end of summer (Douds and Millner, 1999; Oehl et al., 2003). Using successive trap cultures and subsequent extraction and study of spores is time-consuming but may reveal significantly greater diversity (Oehl et al., 2004).

Single-spore inoculation of a suitable host plant allows for establishing monosporic cultures of AM fungi. It can also assist in understanding the individual effects of AM fungi on plant growth and the combined effects of other stresses on different crops. Few studies have been conducted on the propagation of AM fungi from single spores as starter inocula using substrate-based methods (Jansa et al., 2002; Panwar et al., 2007; Selvakumar et al., 2018). We hypothesize that inoculation with AM fungal single spores may help to elucidate the efficiency in preventing the contamination of spores (degraded or parasitized by other organisms) extracted directly from the field. The present study aimed to morphologically characterize AM fungal species associating with maize using single-spore propagation.

## 2 Materials and methods

### 2.1 Soil sampling and treatments

The soils in this study originated from fields (in the Free State and KwaZulu-Natal provinces, South Africa) of different farming practices, including conventional and conservational production of maize and four different treatments: maize after oats (MO), maize after soybean (MS), maize after maize (MM), and maize after peas (MP). Soils were sampled three times, first pre-planting (PP), 2 weeks before planting (October 14, 2019); second germination (GN), 2 weeks after germination (January 9, 2020); and third germination, at harvest (AH) (July 2020). Samples were collected from the bulk (0–10-cm depth) and rhizosphere (0–30-cm depth) soil profiles. From each of the sites [Free State (C, conventional; V, Zunckel farms) and KwaZulu-Natal (Z, Van Rooyenswoning farm)], the upper layer of soil organic matter (SOM) and debris was removed, and the bulk soil was then taken for pre-planting. At the same time, for germination and at harvest, the plant was first removed, and roots with rhizosphere soil were then placed in a sampling bag before the above-ground plant material was removed. Then, an additional bulk soil sample was taken from the vicinity where the plant was removed. Five samples were taken per treatment using an auger, with the aim to spread the intra-field samples as far apart as possible while still being accessible. The samples were then thoroughly mixed to make one composite (a single representative sample, created to reduce some of the massive variability) as described by Okalebo et al. (2002). In the Free State farms (large commercial fields), one soil sample was collected in a center sample, and then four soil samples were collected in a clockwise direction, starting at 9 o’clock, around the center sample when facing away from the farm road. In the KwaZulu-Natal (KZN) fields (under pivot irrigation), soil samples were collected trying to obtain a sample from every side and within every ring left by the irrigation system. Approximately 1 kg of the composite (soil adhering to the roots and next to plants) soil samples were placed in plastic bags (Ziploc freezer bags), labeled, and transported to the Mycorrhizal Laboratory at Rhodes University, Makhanda, Eastern Cape, South Africa, for further processing.

### 2.2 Trap culture

**Figure 1:**
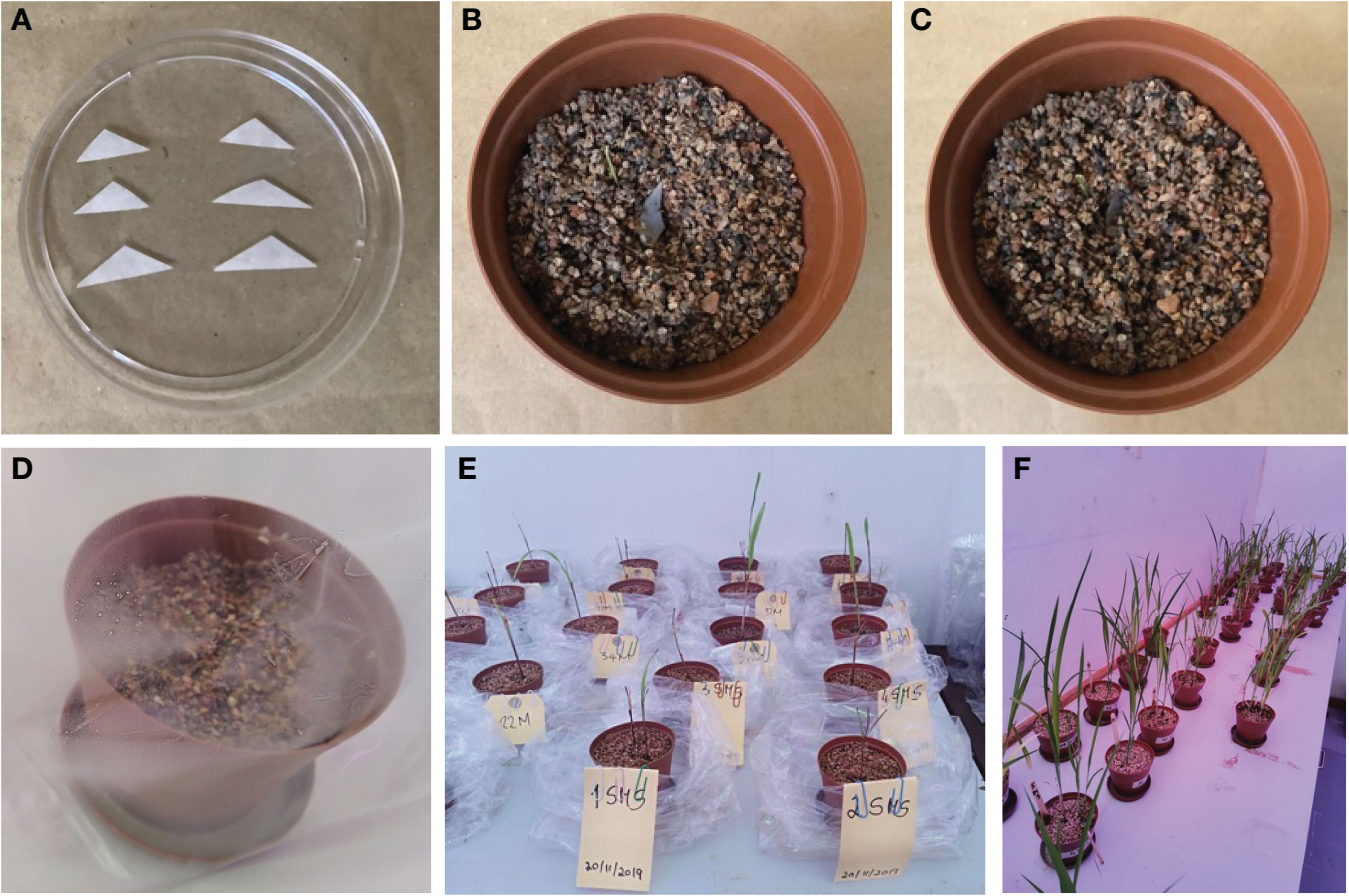
Single spore inoculation method in the pot. **(A)** Triangle filter paper with single spore prepared for inoculation. **(B)** Pre-germinated sorghum inoculated with single spore. **(C)** Inoculated seed covered with the non-clay substrate. **(D)** Single spore inoculated in pre-germinated seedlings. **(E)** pregerminated seedlings. **(F)** Four-month-old seedlings.

AM fungi are obligate biotrophs and cannot be grown in an artificial medium. They must be associated with a host plant. Trap cultures were established to trigger the sporulation of AM fungal species. These cultures aim to maintain a living collection of organisms to study and obtain fresh spores to set up monosporic cultures for identification. *Sorghum bicolor* was chosen as the symbiotic partner because of its high mycorrhizal dependency, wide adaptability, and high resistance to abiotic stresses, including drought, salinity, waterlogging, and heavy metals (Dar et al., 2018). A non-soil clay substrate was sterilized at 121°C for 15 min (Getenga et al., 2004; Kariman et al., 2022) and, when cooled, was placed in 9.5 × 6.5 × 9.5 cm plastic pots (until they were two-thirds filled). A soil sample from the field (5 g each; from four different treatments, MO, MS, MM, MP, and under two different agriculture management practices) was used as inoculum and placed (2 cm deep) in the pots below the seeds (approximately three disinfected seeds of sorghum per pot). More non-soil clay substrate was added until the pot was full. The seeds started to germinate after 3 days. The pots were placed in sunbags (Sigma-Aldrich, St. Louis, MO, USA; B7026) and watered with distilled water (**Figure 1**). Sunbags were sealed and monitored regularly, and water was supplied once a week, if needed, to ensure the soil moisture level was maintained, promote normal growth, and allow for AM fungal infection. Pots were placed in a growth room with external lighting on a 12-h day/night cycle, and the temperature was maintained at 25°C–28°C (Katta, 2016). Pot cultures were grown for 4 months (Oehl et al., 2003), after which the host plant was allowed to dry. After harvesting, the trap contents were thoroughly mixed, substrate and root samples were collected, and spores were extracted as described by Schenck (1982) and Smith and Dickson (1997) and grouped into different morphotypes according to their morphological characteristics observed under a dissecting microscope (Goswami et al., 2018).

#### 2.2.1 Single spore trap culture

Sorghum seeds were surface-disinfected by soaking them in 10% sodium hypochlorite for 30 min and rinsing them three times with sterile distilled water. Using plastic pots (9.5 × 6.5 × 9.5 cm) filled with sterile non-soil clay substrate (autoclaved at 121°C for 15 min), some disinfected sorghum seeds (3 seeds/pot) were sown and grown with regular watering with distilled water in a plant growth room with 12 h of light (25°C) and 12 h of darkness (18°C) (Sreedasyam et al., 2023). The seeds started germinating after 3 days. Following seed germination, a trap culture was established with individual morphotypes by picking up only one spore (that appeared healthy based on shape, color, and surface condition) under a dissecting microscope and transferring it to a small triangular sterilized filter paper (**Figure 1**). Additionally, intact spores of each type were mounted in polyvinyl alcohol/lactic acid/glycerol (PVLG) with (to obtain the most intense staining reaction) and without Melzer’s reagent (to observe diagnostic features with a compound microscope for identification using the keys of Schenck and Perez (1990) and INVAM (http://www.invam.caf.wvu.edu) based on the wall structure and, if possible, to classify the genus and species rank using the current taxonomy (Schüßler and Walker, 2010; Redecker et al., 2013). The tools used here are important in morphology-based diagnoses; they are effective in detecting amyloid A substance present in the spores of some species of fungi that appear as a blue-black stain under the microscope (Josserand 1983; Vizzini et al., 2020). The small filter paper (carrying a single spore) was then carefully inoculated below a root from germinated sorghum seeds (**Figure 1**) in each pot (Brundrett and Juniper 1995; Selvakumar et al., 2018). The spores were then covered with sterile substrate. Cultures with one spore from an existing culture ensure that only one species of fungus is present and can significantly reduce the possibility of transferring parasitic organisms to healthy spores. All pots were placed in sunbags, maintained in a plant growth room (120 days) with regular watering with distilled water, and periodically assessed for AM fungal development. After 4 months, plants were harvested along with complete roots, and spores were extracted for molecular identification of the AM fungal species.

#### 2.2.2 Direct PCR amplification of single spore

Spores obtained from single spore trap cultures from each treatment (MO, MM, MP, and MS) were extracted following the sieving method described by Schenck (1982) and Smith and Dickson (1997). Under a dissecting microscope (Leica L2), a single spore (from each treatment) was picked and transferred to a 1.5-mL centrifuge tube (a different tube for each treatment), and 10 µL of RNAlater™ (Sigma, Lot# MKCP0642) was added to the tube. The tube was left overnight in a refrigerator (4°C), followed by overnight incubation in a freezer (−20°C). The spores were gently crushed in a centrifuge tube using a pipette tip under a stereoscope to ensure the spore wall ruptured. Three pairs of primers were used for PCR amplification: ITS1 × ITS4 (White et al., 1990), SSmAf × LSUmAr/SSUmcf × LSUmBr (Krüger et al., 2009), and NS31 × AML2 (Lee et al., 2008). These primers are known to be suitable for specifically amplifying AM fungi rDNA, characterizing the diversity of AM fungal species, and allowing phylogenetic analysis with species-level resolution. The internal transcribed spacer (ITS) region is the common barcoding region used to identify and distinguish different fungal species (Rajaratnam and Thiagarajan, 2012; Raja et al., 2017). This region includes the ITS1 and ITS2 regions separated by the 5.8S gene, situated between the 18S SSU and 28S LSU genes in the rDNA repeat unit (Wipf et al., 1999). The ITS region is the most used for fungal analysis because it has a higher degree of variation between species that are closely related; this is evident by the sequences on the barcoding gap that show a higher difference in sequences among species than those within species (Toledo et al., 2013; Aslam et al., 2017). The SSU, ITS, and LSU regions have different rates of evolution, which resulted in different levels of genetic variation; e.g., SSU evolves the slowest, resulting in it having the lowest amount of variation among taxa, is highly conserved, and has a high specificity of the primer combination (Lee et al., 2008; Lekberg et al., 2018; Perez-Lamarque et al., 2022). ITS evolves the fastest and shows the highest variation. LSU is generally considered less variable than the ITS region (White et al., 1990; Bruns et al., 1991; Begerow et al., 2010). The greater sequence variation in the ITS (ITS1/ITS2) makes them more suited for species and strain identification than the 18S region, the 5.8S region, and the 28S region (Iwen et al., 2002). PCR was conducted in a total volume of 20 µL consisting of 10 µL of 2x Phire Plant direct PCR Master Mix (Thermo Scientific™, Waltham, MA, USA; Lot# 01098165), 1 µL Primer A, 1 µL Primer B, 6 µL nuclease-free H_2_O, and 2 µL crushed spores in RNAlater. The thermal cycling parameters are presented in **Table 1**.

**Table 1:** Thermal cycle parameters used in the direct PCR amplification.

Parameters	Temperature (°C)	Time (s)	Cycles
Initial denaturation	98	300	1
Denaturation	98	5	35
Annealing (ITS1/ITS4)	49	30	35
Annealing (SSU/LSU)	58	30	35
Annealing (NS31/AML2)	58	30	35
Elongation	72	20	35
Final elongation	72	60	1

Repeated PCR was performed using the same set of primers for ITS1 × ITS4 and NS31 × AML2; however, for SSU and LSU, a new set of primers was used: SSUmcf and LSUmBr (Krüger et al., 2009). PCR amplicons were electrophoresed on a 1% agarose gel and visualized using UV light in a Bio-Rad Molecular Imager^®^ ChemiDoc™ XRS+ with Image Lab™ Software (USA). PCR amplicons were cleaned using a kit from WizardR SV Gel and PCR Clean-Up System (Promega, Madison, WI, USA; ZR-96 DNA Clean-up Kit™). Samples were then sent for Sanger sequencing to Inqaba Biotechnical Industries (Pty) Ltd. in Pretoria.

### 2.3 Microscopy survey

Healthy spores (showing numerous lipid globules inside and neither a turbid content nor an air bubble) obtained from single spore trap cultures and collected by the wet sieving method described by Schenck (1982) and Smith and Dickson (1997) were selected for identification using the morphological approach. The morphological characteristics of the spores and details of the wall structure were determined by examining several slides of intact spores mounted in PVLG (Omar et al., 1979) and a mixture of PVLG and Melzer’s reagent (1:1, v/v). On each labeled microscope slide, two drops of PVLG were added to one set of spores, and one drop of PVLG and one drop of Melzer’s reagent were added to the second set of spores (Koske and Tessier, 1983). A cover slip was placed on each group, and localized light pressure was applied to break the cell walls of some spores. The slides were incubated at room temperature for at least 3–5 days (to clear their contents from the oil droplets) before being examined under a light microscope. After that, they were examined under an Olympus BX50 DIC compound microscope. Microphotographs were recorded using a Sony 3CDD color video camera coupled to a microscope. The terminology for spore structure has been suggested by Stürmer and Morton (1997) and Walker (1983). The spore size and color of fresh specimens immersed in distilled water were examined under a dissecting microscope. Color names were obtained from Kornerup and Wanscher (1983). The number of cell wall layers, the reaction of individual layers with the stain, and the flexibility of the coatings were observed and recorded. The subtending hyphae, continuity of the spore cell walls with those of subtending hyphae, and the existence of a septum or occlusion were also considered. These characteristics were compared to the descriptive features of described species posted on the INVAM website (http://fungi.invam.wvu.edu/the-fungi/species-descriptions.html), and morphotype determination of the genus was made based on the classifications described by Morton and Benny (1990). Each morphotype was assigned the name of the species with the closest match. The nomenclature for fungi is that of Schüßler and Walker (2010) and Redecker et al. (2013)

### 2.4 Data analysis

Nucleotide sequences obtained from Sanger sequencing were aligned on MEGA X version 10.2.4 (Kumar et al., 2018; Stecher et al., 2020) using MUSCLE (Edgar, 2004a, 2004b). MEGA X and MUSCLE settings were by default for gap penalties and memory/iterations, while advanced options were by the neighbor-joining method. The fasta file containing the nucleotide sequences was subjected to a Basic Local Alignment Search Tool (BLAST^+^) program available at the National Center for Biotechnology Information (NCBI) website (http://www.ncbi.nlm.nih.gov) for comparison against the GenBank database (Altschul et al., 1997) to determine the closest sequence matches that enabled taxonomic identification. The most comparable sequence matches with at least 97% similarity (this threshold is considered given rather than being a tunable parameter, following the conventional wisdom that 97% corresponds approximately to species; Schloss and Handelsman, 2005; Westcott and Schloss, 2017) with the reference sequence, and meant taxonomic identification to the genus level could be selected. The nomenclature of AM fungal genera was assigned according to the Index Fungorum website (https://www.indexfungorum.org) to determine the currently accepted name. Additional taxonomic assignment was based on phylogenetic relationships. A phylogenetic tree was constructed based on multiple sequence alignments from sequences of AM fungal isolates (in the four treatments: MM, MO, MP, and MS) and GenBank (NCBI) data sequences and was estimated by the neighbor-joining method (Saitou and Nei, 1987) using unbiased estimates of evolutionary distances. The bootstrap tests assessed the reliability for the maximum parsimony (Kumar et al., 2018). The branches corresponding to partitions reproduced in less than 50% of bootstrap replicates collapsed (Felsenstein, 1985). *Chrysoporthe austroafricana* (JN942337 and JN942338) sequences were taken from the NCBI database and used as an outgroup.

## 3 Results

### 3.1 Single spore identification

**Figure 2:**
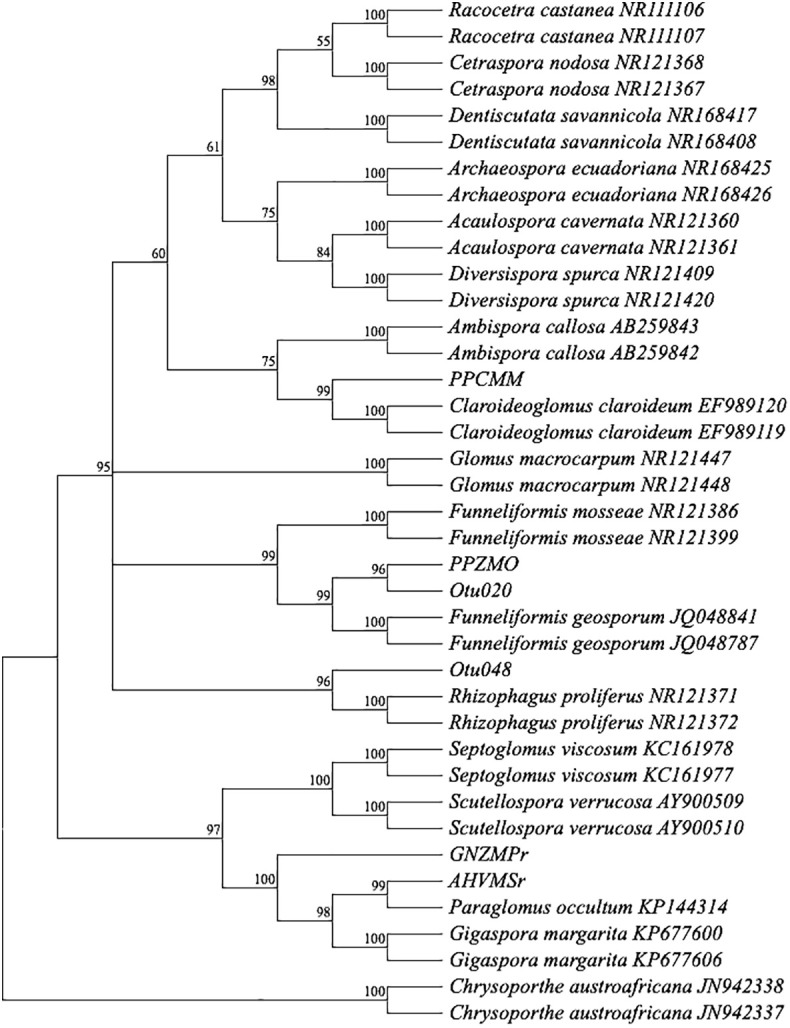
Bootstrap consensus tree. A neighbor-joining phylogram of selected sequences of AM fungi based on distance analysis of the 18S rDNA. For comparison, identified sequence types from GenBank were included in the analysis. Bootstrap supports values greater than 50%, given on the branches (1,000 resembling branches). Samples from this study do not show an accession number. The tree was rooted in *Chrysoporthe austroafricana*. PP, pre-planting; GN, germination; AH, at harvest; r, rhizosphere; MO, maize after oats; MM, maize after maize; MP, maize after peas; MS, maize after soybean; Z, Zunckel farm; C, conventional farm; V, Van Rooyenswoning farm; AM, arbuscular mycorrhiza.

After 4 months of observation of 32 inoculants (trap culture pots inoculated with single spores from each treatment) in the plant growth room, only 29 inoculants showed propagations with more than 100 spores (**Figure 1**). Out of the 29 propagations, only 13 samples had good sequence (Sanger) quality, and one sample (good chromatogram with high-quality peaks) from each treatment was chosen for further analysis. BLAST^+^ on NCBI was performed for ID identification. The analysis involved 39 nucleotide sequences. All ambiguous positions were removed for each sequence pair (pairwise deletion option). There were a total of 1,259 positions (ITS1 × ITS4) in the final dataset (Kumar et al., 2018; Stecher et al., 2020). The percentage of replicate trees in which the associated taxa clustered together in the bootstrap test (1,000 replicates) is shown next to the branches (**Figure 2**) (Felsenstein, 1985). The constructed phylogenetic tree separated the four AM fungal isolates from their outgroup, *C. austroafricana*. This phylogenetic tree (**Figure 2**) showed that the PPCMM isolates formed the same cluster as *Claroideoglomus claroideum* and were in a different clade from the *Ambispora* genus. The AHVMSr isolates formed the same cluster with the *Paraglomus* genus and on a distinct clade with *Gigaspora margarita*. The phylogenetic tree also showed that the GNZMPr isolate was in the same clade as *G. margarita* and *Paraglomus occultum*. The PPZMO isolate was in the same clade as *Funneliformis geosporus*. In the phylogenetic tree, the closest neighbors of the propagated AM fungal species revealed that the AM fungal spores had characteristics belonging to the genera *Claroideoglomus* (PPCMM), *Gigaspora* (GNZMPr), *Paraglomus* (AHVMSr), and *Funneliformis* (PPZMO) (**Figure 2**) and were present under both agriculture practices. Also, described species such as OTU024 and OTU021 could be seen in the tree close to the genera *Funneliformis* and *Rhizophagus*.

### 3.2 Morphological characterization of AM fungi

**Figure 3:**
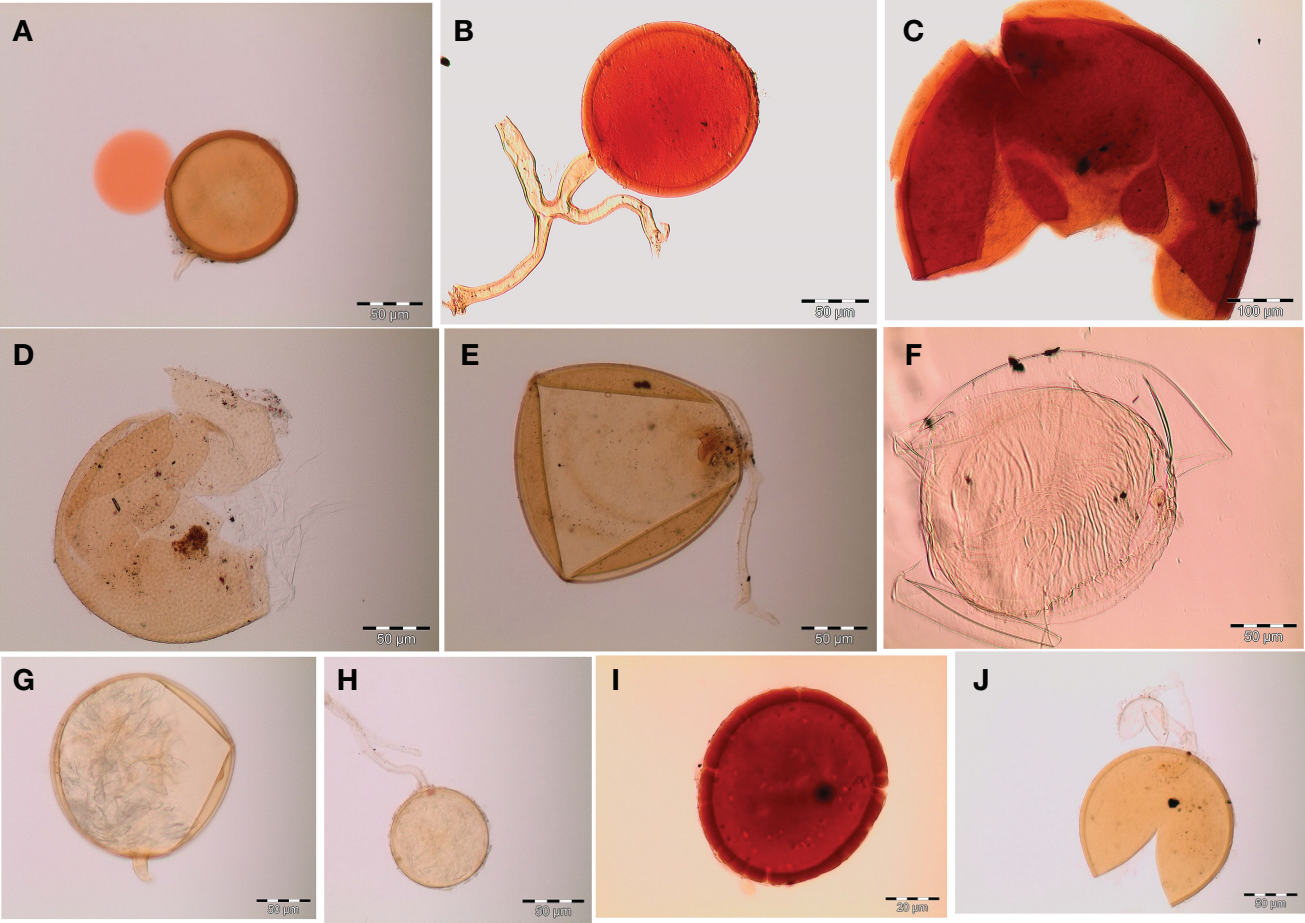
Spore identification using morphological characteristics: **(A, B)** *Funneliformis mosseae*, **(C)** *Gigaspora margarita*, **(D)** *Acaulospora cavernata*, **(E)** *Rhizophagus intraradices*, **(F)** *Diversispora spurca*, **(G, H)** *Glomus macrosporum*, **(I)** *Funneliformis geosporus*, and **(J)** *Paraglomus occultum*.

Spores (some) obtained from the single spore trap cultures were used for morphological characterization. The macro-characteristics considered for description included the spore color, size, and shape; shape, width, pore occlusion of the subtending hypha; and the size of the auxiliary cells. The micro-characteristics for characterizing the spore wall structure included color, dimension, number, type, and ornamentation (**Table 2**). Yellowish, reddish brown, dark red-brown, yellow-brown, and hyaline to pale yellow spores were observed in this study, with sizes ranging from 128- to 220-µm diameters. Overall, eight species of AM fungi belonging to five families in the Glomeromycota phylum were found in this study: Acaulosporaceae (one species), Diversisporaceae (one species), Gigasporaceae (one species), Glomeraceae (four species), and Paraglomeraceae (one species) (**Figure 3**; **Table 2**). Furthermore, from a total of 10 morphotypes of AM fungal generated and classified on Mothur using the UNITE database, the results were then blasted on NCBI for comparison. Sequences were deposited into GenBank under accession numbers OR822200–OR822205. Species such as *Acaulospora lacunosa*, *Archaeospora leptoticha*, *G. margarita*, *Gigaspora rosea*, *Funneliformis mosseae*, *F. geosporus*, *Glomus monosporum*, *Rhizophagus aggregatus*, *Rhizophagus intraradices*, and *P. occultum* showed more than 97% similarity with NCBI comparison (**Supplementary Table 1**).

**Table 2:** Morphological characteristics of some AM fungal species.

AM fungal species	Description
*Acaulospora cavernata* Blaszk (1989) spores in PVLG 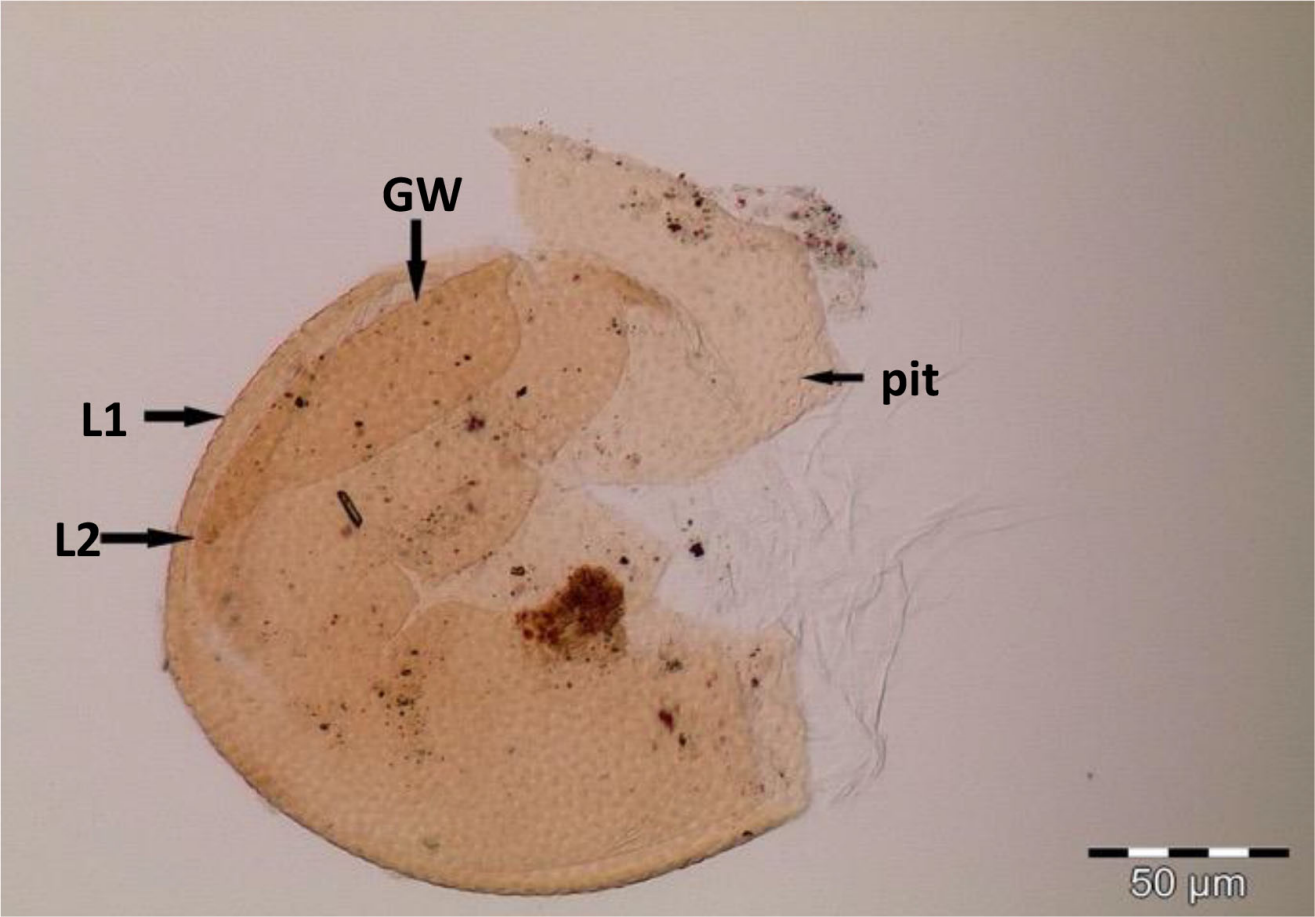	Spore pale yellowish in color to light brown with two spore wall layers (L1 and L2), namely, outer (L1) membranous light yellow colored layer. Sub-globose, 130–150 µm in diameter, sessile on the neck of a sporiferous saccule. Mature spore showing one terminal germinal wall (GW) layer and pitted ornamentation on the wall
*Funneliformis geosporus* C. Walker & A. Schüßler (2010) spores in PVLG + Melzer’s reagent 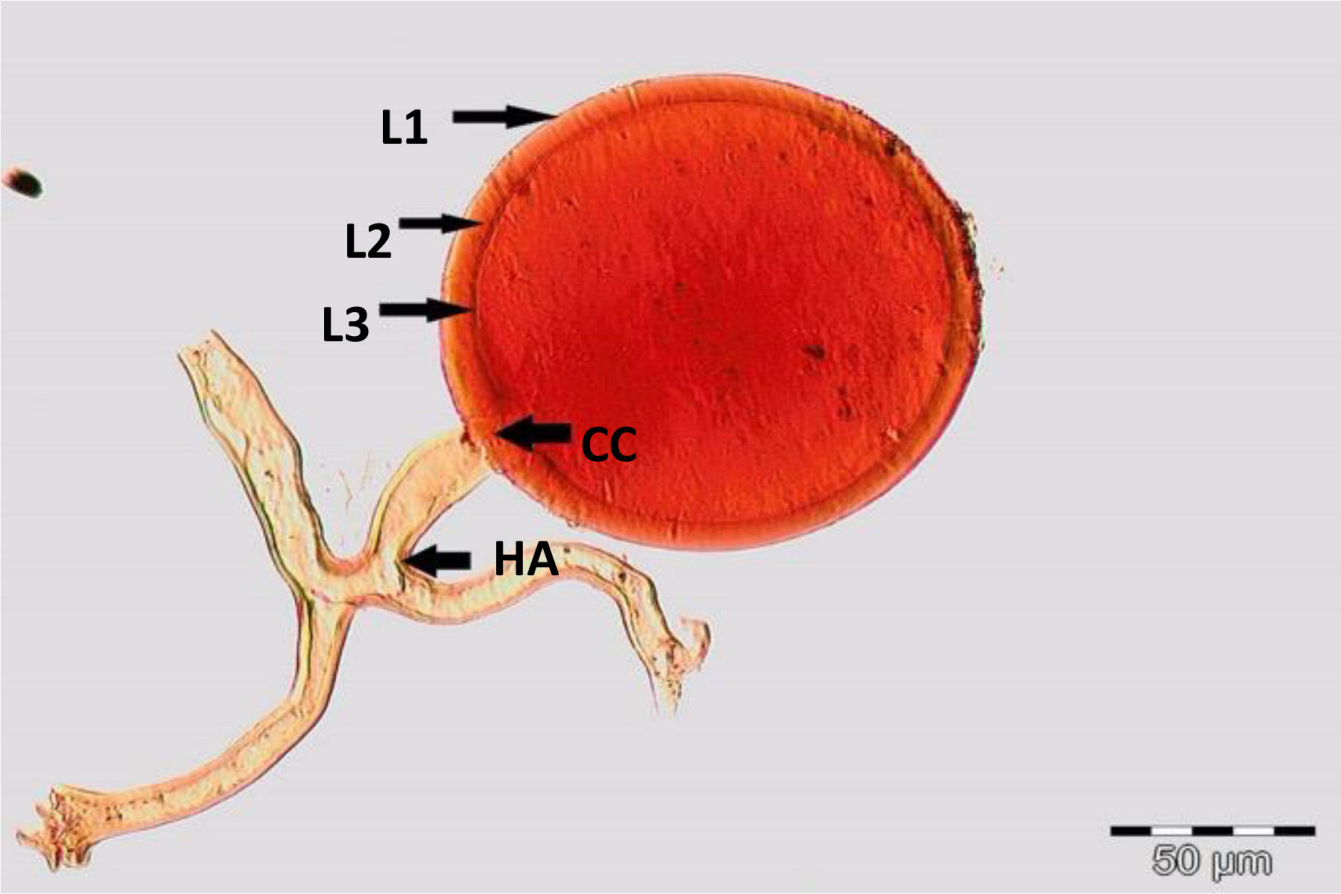	Reddish brown walled spore, with lengthy tube-like subtending hyphae. Spore shows three wall layers (L1, L2, and L3), which are tightly adherent, laminated, and membranous. L1, a hyaline sloughing granular layer; L2, a rigid layer consisting of adherent sublayers appeared orange-brown in color; L3, a semi-rigid resolved by slightly darker color (yellow to orange-brown). A germ tube emerged from the lumen of the subtending hypha and originated from the recurved septum. Globose, 128–135 µm in diameter with loosely sleeve-like hyphal attachment at right angle to spore wall. Cross channel (CC) in wall layers, common hyphal attachment (HA)
*Gigaspora margarita* W.N. Becker & I.R. Hall (1976) spores in PVLG + Melzer’s reagent 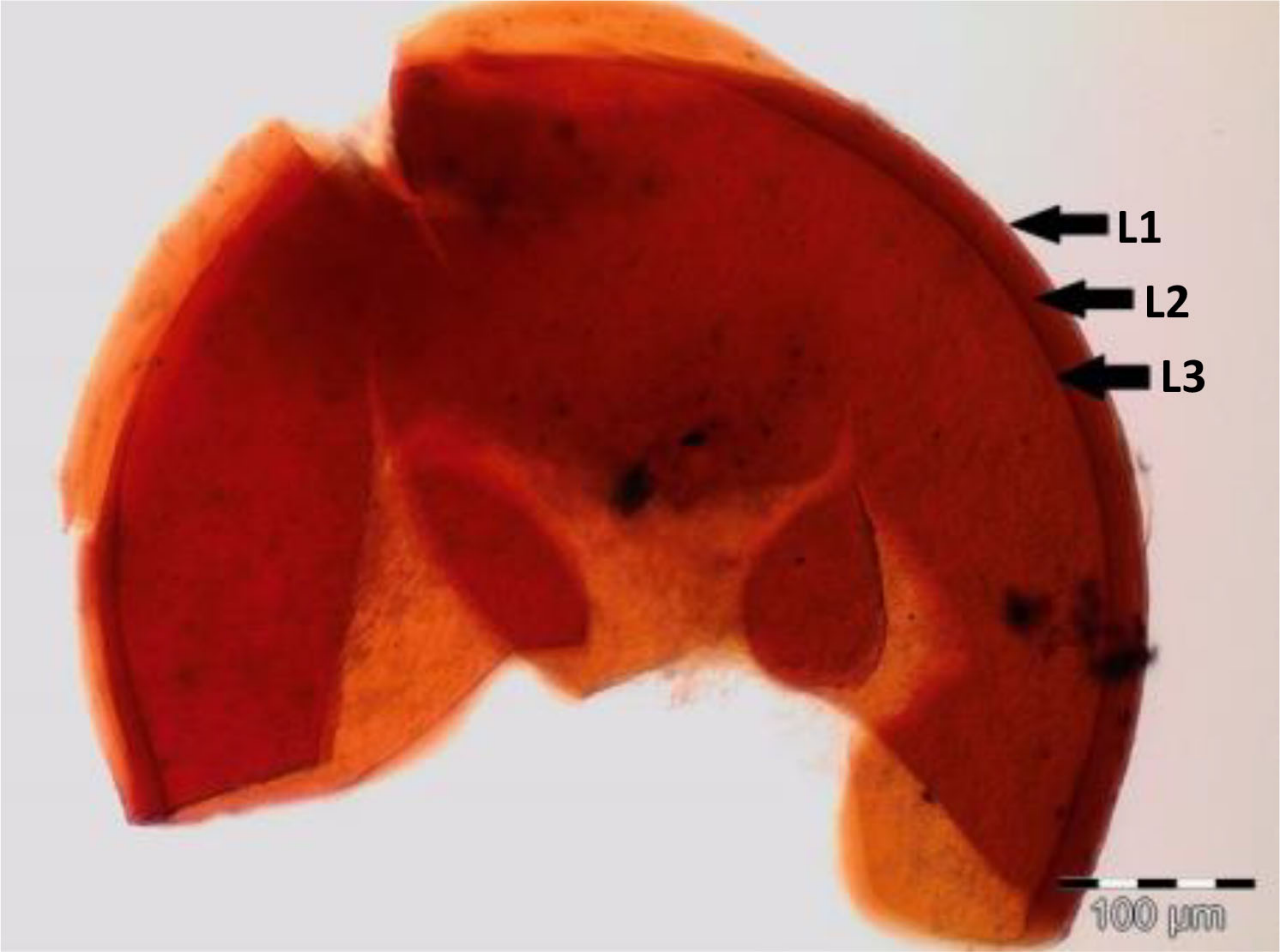	Crushed spore of *Gigaspora margarita*, globose in shape, dark red-brown in color, and consisted of three layers: L1, an outer permanent rigid layer, smooth, adherent to inner laminae, pale brownish; L2, hyaline sublayer, rigid, dark red-brown; and L3, germinal layer that is concolorous and adherent with the laminate layer, 215–220 µm in diameter
*Diversispora spurca* C. Walker & A. Schüßler (2004) spores in PVLG 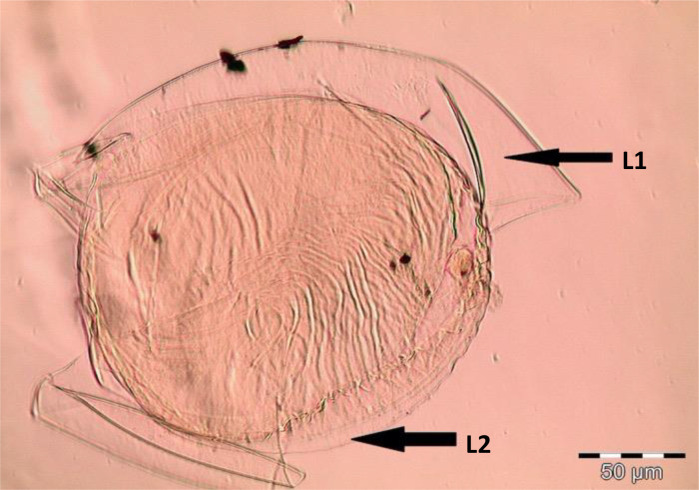	Crushed spore of *Diversispora spurca*, subhyaline, globose in shape, and consisted of two layers: L1, hyaline to pale yellow-brown, separating from the L2; and L2, thin hyaline to subhyaline sublayer. L2 of the spore wall stops abruptly in the region of attachment and thus is not part of the more distant hyphal wall structure; 155–182 µm in diameter
*Rhizophagus aggregatus* in PVLG + Melzer’s reagent 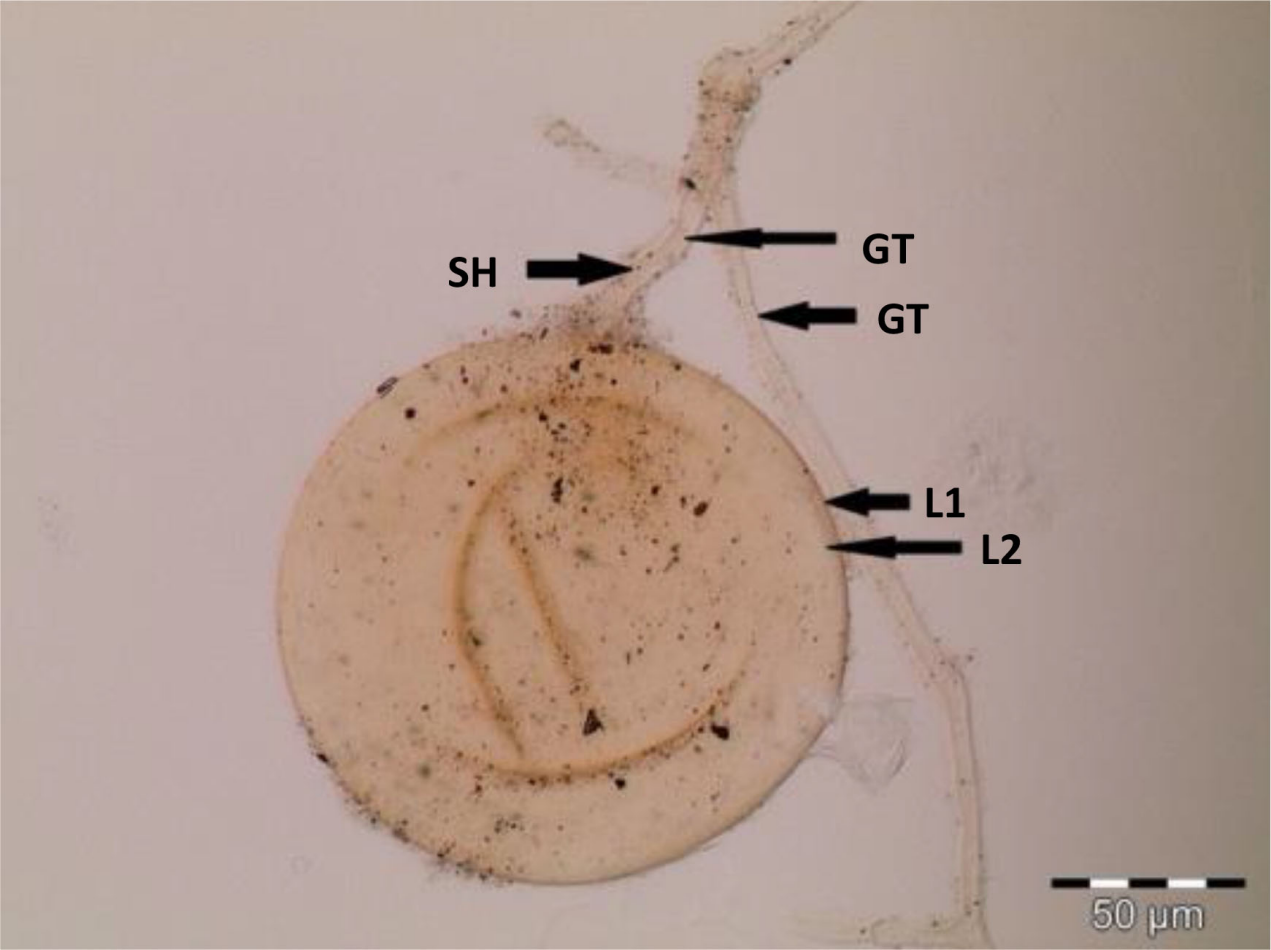	Globose spores of *R. aggregatus*, 143–145 µm in diameter, hyaline to pale yellow in color, with two yellow layers (L1 and L2). Single subtending hyphae, which stop abruptly in the region of attachment and thus are not part of the more distant hyphal wall structure. The hypha is closed by a thin septum, thickening of the spore wall. Germ tube (GT), subtending hyphae (SH)
*Paraglomus occultum* (C. Walker) J.B. Morton & D. Redecker (2001) spores in PVLG 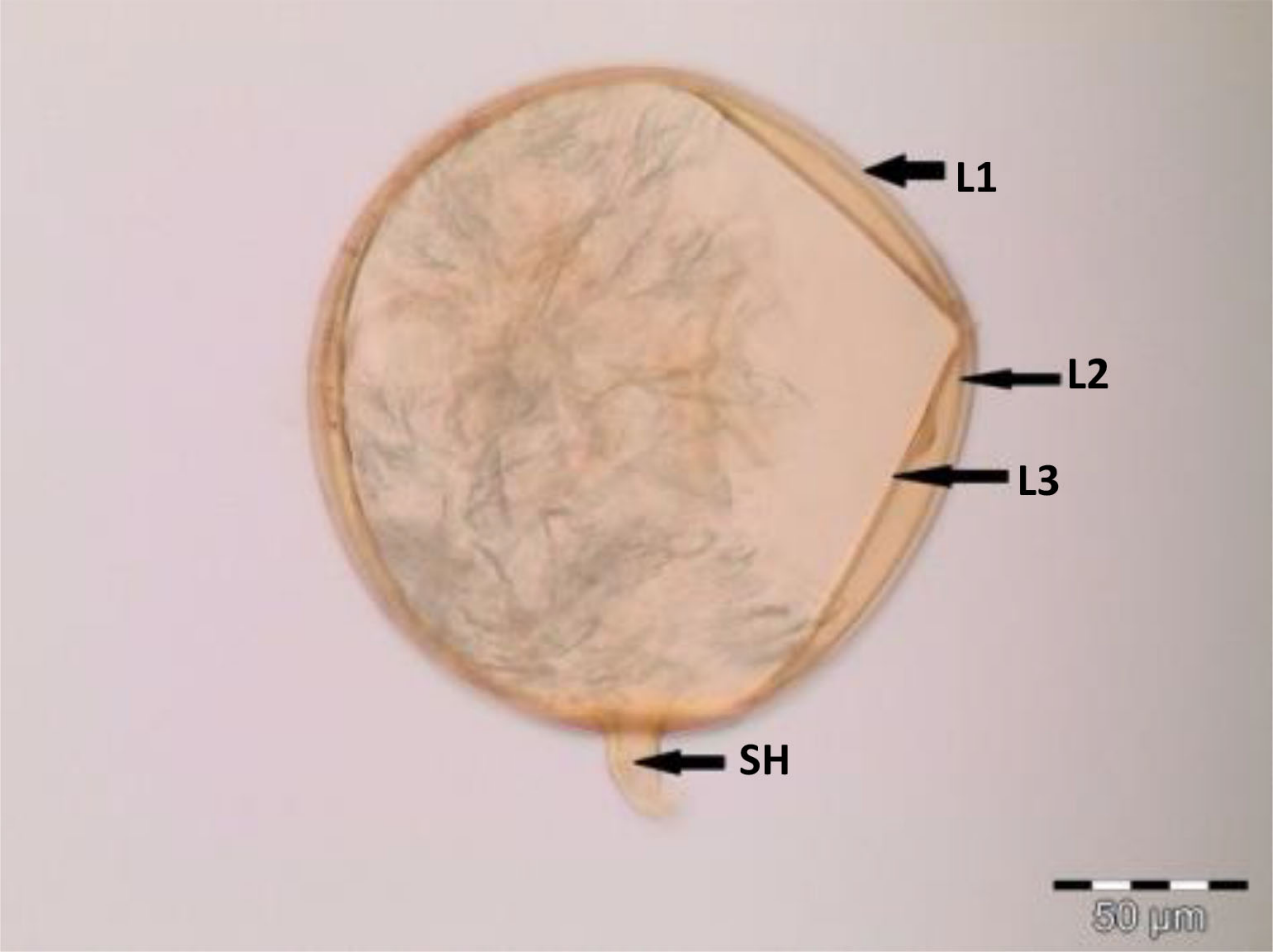	Globose spore of *Paraglomus occultum*, 152–159 µm in diameter, hyaline to pale yellow in color, with three layers (L1, L2, and L3). L1, a sloughing layer; L2, a permanent layer, continuing into the wall of subtending hypha; and L3, a permanent layer, SH subtending hyphae
*Funneliformis mosseae* C. Walker & A. Schüßler (2010) spores in PVLG + Melzer’s reagent 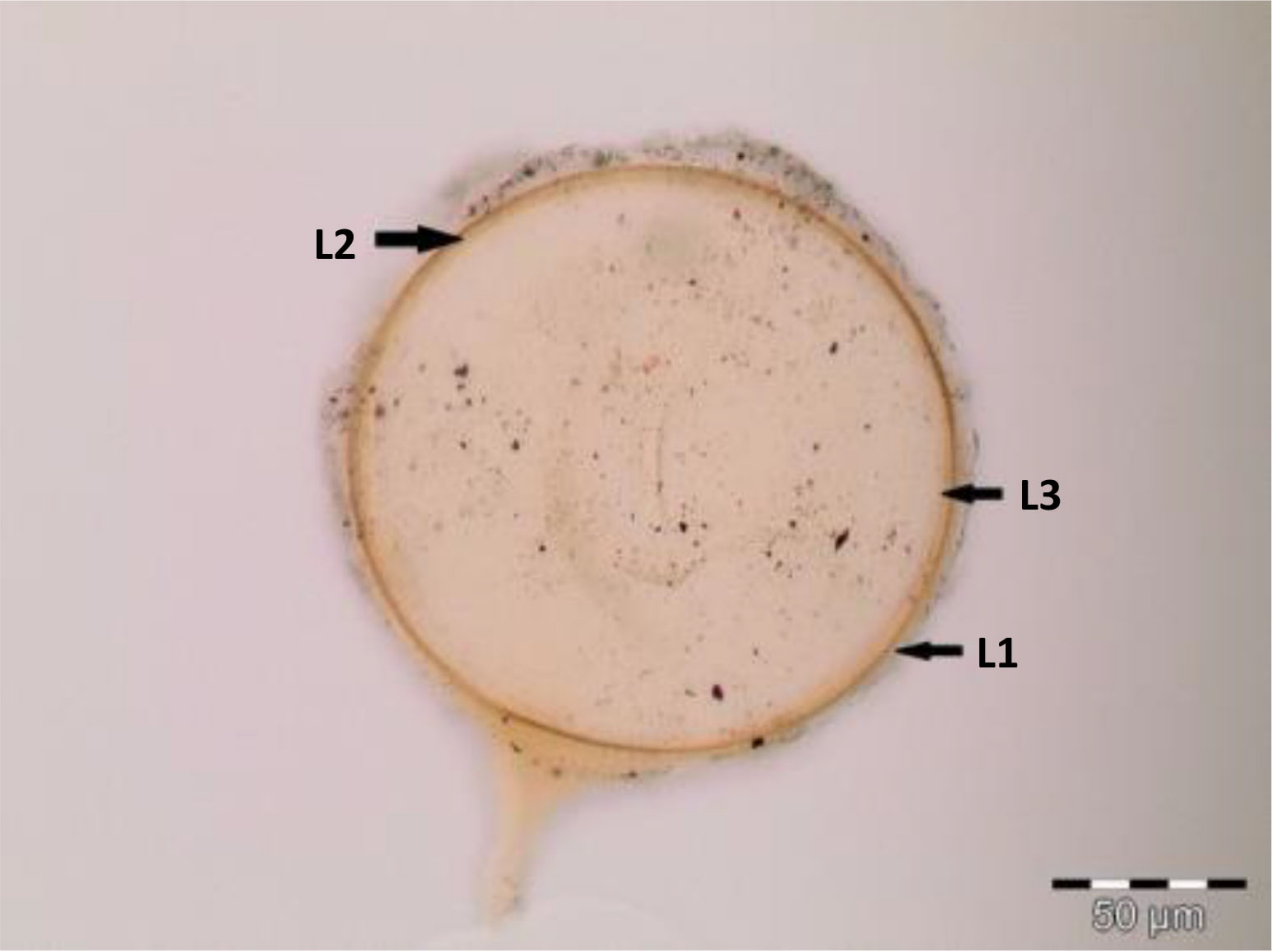	Globose spore of *Funneliformis mosseae*, 152–154 µm in diameter, hyaline to straw to yellow in color, with three layers (L1, L2, and L3). L1 hyaline, mucilaginous; L2 hyaline, rigid, attached firmly to the underlying laminae; and L3 pale sublayer

AM, arbuscular mycorrhiza; PVLG, polyvinyl alcohol/lactic acid/glycerol.

## 4 Discussion

Trap culture and monosporic culture techniques of spore multiplication are the most commonly employed cultivation strategies in the substrate-based method (Douds et al., 2006; Panwar et al., 2007). These two AM fungal propagation techniques provide an environment that closely mimics the field conditions. Since AM fungi have been reported to be able to provide their hosts with access to numerous soil resources such as P, N, and water, the present study was involved in the propagation of AM fungal single spores isolated from maize soil samples from four different treatments (PPZMO, AHVMSr, PPCMM, and GNZMPr) under two different agriculture management practices. The initial inoculum should start with a single spore to obtain a pure culture. Only 29 samples out of the initial 32 pots showed some colonization in this study. These could be because of the source of the initial AM fungal spores or the quality of the spores, as the spores were isolated from soil samples under different crop rotations, which could have influenced the quality of the spores. Maize is an obligatory mycorrhizal species readily colonized by many non-host-specific AM fungi (Daisog et al., 2012). Maize growth phases strongly affect the abundance, diversity, and community composition of AM fungi (Lü et al., 2020). In the current study, the selected single spores were picked from soil samples collected in different farms and different maize growth phases (PP, AH, and GN), which could explain the occurrence of different AM fungal species at a particular stage of maize development, underlying the fact that AM fungi have contrasting seasonal sporulation dynamics and their different phenologies can cause disparate community composition across plant growing seasons (Pringle and Bever, 2002; Bainard et al., 2012; Varela-Cervero et al., 2016). Also, Mathimaran et al. (2007) and Nord and Lynch (2009) demonstrated that plant phenologies and the dynamics of soil processes could likely differ in different cropped soils.

Identification of AM fungi both morphologically and molecularly is essential to determine with some certainty the identity of an organism (Schenck and Perez, 1990; Lee et al., 2006; Schüßler and Walker, 2010). Brundrett (1991) suggested a variation of AM fungal species in a habitat, which is related to their capability of growing under certain conditions and the ability of fungi to form a symbiosis with various types of plants in the vicinity. Identification of the four AM fungal isolates based on morphology complemented the identification based on molecular (species level) analysis. Therefore, there are more detailed and valid data, high confidence, and strong evidence that the morphological characterization technique is also important for AM fungal identification. Nonomura et al. (2011) stated that combining the results from morphological characterization and molecular methods is the best approach to identifying AM fungal taxonomy. Also, spores are viewed as among the most important and convenient characteristic features, and they can help researchers rapidly identify mycorrhizae faster than sequence techniques (Fall et al., 2022). In this study, AM fungal species belonging to the genera *Glomus*, *Funneliformis*, *Gigaspora*, *Acaulospora*, *Diversispora*, *Rhizophagus*, and *Paraglomus* were associated with maize and described morphologically. Similar results were expected in the work of Tobolbai et al. (2018); Baltruschat et al. (2019), and Fall et al. (2022) where they studied the morphological diversity of native AM fungal species associated with the rhizosphere of maize in different agroecosystems and found similar AM fungal species. Also, studies by Sanders (2003); Hijri et al. (2006); Alguacil et al. (2008), and Sasvári et al. (2011) described the seven genera (similar to the ones encountered in the current study) as the most dominant genera associated with maize crops under different management practices.

The applications of the findings of this research, particularly for the maize crop, show that all AM fungal spores obtained are good candidates for mass-producing inocula or material for identification in other studies and, also, can guide the management of these important symbioses as part of integrated conservation of land management plans.

## 5 Conclusion

The results of the current study demonstrated that the production of pure cultures of AM fungal species could be achieved using single-spore inoculum through the trap culture method. Selected AM fungal isolates (from the most predominant spore morphotypes) exhibited more spores produced, and all belonged to the phylum Glomeromycota. Future studies are needed to further identify more different genera in the Glomeromycota to explore the diversity of AM fungal species associated with maize and, if possible, authenticate the method applied in this study. This applied method can also be used to initiate starter cultures for bioinoculants in large-scale agricultural applications with the advantage of being more readily adopted by farmers due to the lack of requirement of a skilled technique in spore propagation.

## Data availability statement

The original contributions presented in the study are included in the article/**Supplementary Material**. Further inquiries can be directed to the corresponding author.

## Author contributions

SM-S: Conceptualization, Formal analysis, Methodology, Writing – original draft. JD: Conceptualization, Methodology, Supervision, Writing – review & editing.
