# Supplementary material for: Characterization of arbuscular mycorrhizal fungal species associating with Zea mays
Source: Front Plant Sci. 2024 May 7;15:1345229. doi: 10.3389/fpls.2024.1345229 (PMC11106459; doi:10.3389/fpls.2024.1345229)
Supplement: Supplementary file 1 [file Table_1.docx]

Supplementary Table 1. AM fungi species occurrence in each sample based on the UNITE database.

| **Family** | **Genus** | **Species** | **PPZMP** | **PPCMM** | **PPVMS** | **PPZMO** | **GNZMPr** | **GNZMOr** | **GNVMSr** | **GNCMMr** | **AHZMPr** | **AHZMOr** | **AHVMSr** | **AHCMMr** | **AHVMS** | **AHCMM** |
| --- | --- | --- | --- | --- | --- | --- | --- | --- | --- | --- | --- | --- | --- | --- | --- | --- |
| Acaulosporaceae | *Acaulospora* | *lacunosa* | 0 | 1 | 0 | 1 | 0 | 0 | 0 | 0 | 0 | 0 | 0 | 1 | 1 | 0 |
|  |  | sp. | 0 | 0 | 0 | 0 | 0 | 0 | 1 | 0 | 0 | 0 | 1 | 0 | 0 | 0 |
| Ambisporaceae | *Ambispora* | *callosa* | 1 | 0 | 1 | 0 | 1 | 1 | 0 | 0 | 0 | 0 | 1 | 0 | 1 | 0 |
|  |  | sp. | 1 | 0 | 1 | 0 | 1 | 0 | 0 | 0 | 0 | 0 | 1 | 1 | 1 | 1 |
|  |  | *leptoticha* | 0 | 0 | 1 | 0 | 1 | 0 | 0 | 0 | 0 | 0 | 1 | 1 | 1 | 1 |
| Archaeosporaceae | *Archaeospora* | sp. | 1 | 0 | 0 | 0 | 0 | 0 | 0 | 0 | 0 | 0 | 0 | 0 | 0 | 0 |
| Gigasporaceae | *Gigaspora* | *margarita* | 0 | 0 | 1 | 0 | 1 | 0 | 1 | 0 | 1 | 0 | 0 | 1 | 1 | 0 |
|  |  | sp. | 0 | 0 | 1 | 0 | 1 | 0 | 1 | 0 | 1 | 0 | 0 | 1 | 1 | 1 |
| Glomeraceae | *Funneliformis* | *mosseae* | 1 | 1 | 1 | 1 | 1 | 0 | 0 | 0 | 1 | 1 | 0 | 0 | 0 | 0 |
|  |  | sp. | 1 | 0 | 1 | 1 | 1 | 0 | 0 | 0 | 1 | 1 | 0 | 0 | 0 | 0 |
|  | *Glomus* | sp. | 1 | 1 | 1 | 1 | 1 | 1 | 1 | 1 | 1 | 1 | 1 | 1 | 1 | 1 |
|  | *Rhizophagus* | *clarus* | 0 | 0 | 0 | 1 | 0 | 0 | 0 | 0 | 0 | 0 | 0 | 1 | 0 | 1 |
|  |  | *irregularis* | 1 | 0 | 1 | 0 | 0 | 0 | 0 | 0 | 1 | 0 | 1 | 0 | 1 | 0 |
|  |  | sp. | 1 | 1 | 1 | 1 | 0 | 0 | 0 | 0 | 1 | 1 | 1 | 0 | 1 | 0 |
| Paraglomeraceae | *Paraglomus* | *occultum* | 0 | 0 | 0 | 0 | 0 | 0 | 0 | 0 | 0 | 1 | 1 | 1 | 1 | 1 |
|  |  | sp. | 0 | 0 | 0 | 0 | 0 | 0 | 0 | 0 | 0 | 0 | 1 | 1 | 1 | 1 |

^0^ no species occurrence

^1^ species occurrence
